# Cryptogenic stroke associated with low ADAMTS13 activity without microangiopathic hemolytic anemia: a comparative study of clinical features and outcomes

**DOI:** 10.1016/j.rpth.2026.106998

**Published:** 2026-10-01

**Authors:** Zhengrui Xiao, Yu-Chiang Chang, Johanna Seiden, Roha Saeed Memon, Simo Du, Parth Patel, Ana Yasmin Caceres Lessa, Gretchen Mackie, Michael Brodsky, Charles Esenwa, Ellen W. Friedman, Irina Murakhovskaya

**Affiliations:** 1 Division of Hematology, Department of Hematology-Oncology, Montefiore Medical Center, Albert Einstein College of Medicine, Bronx, New York, USA; 2 Department of Medicine, Danbury Hospital, Danbury, Connecticut, USA; 3 Department of Neurology, Montefiore Medical Center, Albert Einstein College of Medicine, Bronx, New York, USA; 4 Department of Medicine, Jacobi Medical Center, Albert Einstein College of Medicine, Bronx, New York, USA; 5 Department of Hematology-Oncology, Montefiore Medical Center, Albert Einstein College of Medicine, Bronx, New York, USA

Letter to the Editor

Severe deficiency of ADAMTS13 (a disintegrin and metalloproteinase with thrombospondin 1 motif, member 13) results in impaired cleavage and accumulation of highly procoagulant high-molecular-weight von Willebrand factor multimers. These ultralarge multimers promote platelet-rich microthrombus formation within the microvasculature, compromising blood flow to the brain and other vital organs. Consequently, patients may develop a spectrum of neurologic manifestations, including focal neurologic deficits, seizures, and coma [1]. Low ADAMTS13 activity has been associated with an increased risk of ischemic stroke in a large population-based cohort study [2]. More than 10% of the immune thrombotic thrombocytopenic purpura (iTTP) survivors develop ischemic stroke during follow-up, which correlates with reduced ADAMTS13 activity [3]. Several case series have described patients in whom reduced ADAMTS13 activity was implicated as a potential cause of ischemic stroke in the absence of microangiopathic hemolytic anemia (MAHA) [4], [5]. However, the association between low ADAMTS13 activity and stroke remains poorly understood, and there is currently no consensus regarding optimal management. Therefore, we conducted a retrospective study to characterize patients presenting with stroke and reduced ADAMTS13 activity in the absence of MAHA.

We identified patients using the International Classification of Diseases, 10th Revision diagnosis code for thrombotic thrombocytopenic purpura (TTP) (M31.1) and extracted clinical data from the electronic medical records at Montefiore Medical Center between January 2010 and October 2025. Patients were included if they had an ADAMTS13 activity of ≤20%, consistent with the international TTP Working Group Consensus definition of ADAMTS13 relapse in TTP survivors without clinical recurrence [6]. The absence of MAHA was defined by normal platelet and reticulocyte counts, normal haptoglobin, a lactate dehydrogenase (LDH) level less than 1.5 times the upper limit of normal, and less than 1% of schistocytes (less than 2 schistocytes per high-power field) on a peripheral blood smear [7]. Cryptogenic ischemic stroke was defined by a neurologist when no alternative etiology for stroke was identified after standard diagnostic evaluation. Patients with other potential etiologies of stroke, including atrial fibrillation, endocarditis, or positive antiphospholipid antibodies, were excluded. TTP survivors with laboratory ADAMTS13 relapse in the absence of clinical recurrence served as the control group. Baseline demographic and clinical characteristics, laboratory results, treatments, and patient outcomes were collected and summarized descriptively and compared between the stroke and control groups. Categorical variables were analyzed using the chi-squared test, and continuous variables were compared using the Mann–Whitney *U*-test for small samples.

A total of 63 patients with a diagnosis of iTTP were identified. Ten patients met the inclusion criteria for the stroke cohort, with one experiencing 2 separate stroke episodes, resulting in a total of 11 stroke events. The control group consisted of 9 iTTP survivors with laboratory ADAMTS13 relapse without clinical recurrence, accounting for 14 episodes of laboratory ADAMTS13 relapse. In the stroke group, 7 stroke events occurred in patients with a prior history of iTTP, and 4 events had no previous diagnosis of iTTP. For patients with a prior history of iTTP, the median time from the last clinical iTTP event to the onset of stroke was 11 years.

The median age at stroke in the stroke cohort was 46 years; the majority were female (9/11), and all were from minority populations (4 Hispanic, 7 non-Hispanic Black patients). Most stroke patients had preexisting vascular risk factors, including hypertension (73%), diabetes mellitus (36%), hyperlipidemia (27%), cardiac disease (45%), and prior history of stroke (64%).

Among the 11 stroke episodes, 10 involved the middle cerebral artery, and 1 was a cerebellar infarct. ADAMTS13 activity was less than 3% in 9 episodes, while in the remaining 2 episodes, activity was 14% and 17%, respectively. All episodes were associated with the presence of an ADAMTS13 inhibitor, consistent with immune-mediated TTP.

Compared with the control group, the stroke group had a significantly higher proportion of non-Hispanic Black patients (63.6% vs 7.1%, *P* = .007), higher prevalence of hypertension (72.7% vs 7.1%, *P* = .002), cardiac comorbidities (45.5% vs 0%, *P* = .009), and prior stroke (63.6% vs 7.1%, *P* = .007) (Table 1). Patients in the stroke group also had significantly higher absolute neutrophil counts, LDH, serum creatinine, and blood urea nitrogen, whereas platelet counts were significantly lower compared with the control group. There were no significant differences between the groups with respect to hemoglobin, absolute reticulocyte count, haptoglobin, indirect bilirubin, liver enzymes, ADAMTS13 activity, or ADAMTS13 inhibitor titers (Table 2).

**Table 1:** Baseline characteristic for stroke and control group.

	Stroke group (*N* = 11)	Control group (*N* = 14)	*P* value
**Baseline characteristics**			
**Age**	46 (40-60)	42.5 (30-52.3)	.161
**Gender**			
**Female**	9	9	.407
**Ethnicity**			
**Non-Hispanic White**	0	5	**.046**
**Non-Hispanic Black**	7	1	**.007**
**Hispanic**	4	8	.428
**Body mass index**	38.5 (26.9-40.6)	32.8 (27.8-35.6)	.292
**Comorbidities**			
**Hypertension**	8	1	**.002**
**Diabetes mellitus**	4	1	.133
**Hyperlipidemia**	3	2	.623
**Cardiac disease**	5	0	**.009**
**Coronary artery disease**	2	0	.183
**Heart failure**	1	0	.440
**Myocarditis**	2	0	.183
**Cerebral vascular disease**	7	1	**.007**
**Ever smoker**	6	3	.115
**Former smoker**	5	3	.389
**Active smoker**	1	0	.440

Continuous variables are summarized as median and interquartile range (25th-75th percentiles).

Bold values indicate *P* < .050.

**Table 2:** Laboratory results for stroke and control groups.

	Stroke group (*N* = 11)	Control group (*N* = 14)	*P* value
**Laboratory data**			
**Hemoglobin (g/dL)**	12.9 (11.8-13.9)	13.4 (13.0-14.9)	.092
**Absolute reticulocyte count (k/μL)**	71.6 (54.0-105.3)	65.5 (48.8-72.3)	.229
**Absolute neutrophil count (k/μL)**	5.7 (3.9-6.5)	3.7 (3.1-4.0)	**.017**
**Platelet count (k/μL)**	186.0 (137.0-255.0)	289.0 (215.0-342.5)	**.015**
**Absolute lymphocyte count (k/μL)**	2.1 (1.5-3.5)	2.2 (1.4-3.0)	.840
**Neutrophil-to-lymphocyte ratio**	2.2 (1.6-3.4)	1.7 (1.1-2.5)	.153
**Haptoglobin (mg/dL)**	145.0 (82.0-205.0)	63.5 (13.5-222.3)	.343
**Lactate dehydrogenase (U/L)**	283.0 (221.0-323.0)	204.5 (174.5-225.0)	**.015**
**Blood urea nitrogen (mg/dL)**	17.0 (13.0-21.0)	12.0 (11.0-13.0)	**.014**
**Creatinine level (mg/dL)**	0.93 (0.87-1.15)	0.81 (0.79-0.86)	**.023**
**Aspartate aminotransferase (U/L)**	25.0 (20.0-31.0)	22.0 (20.0-25.8)	.566
**Alanine aminotransferase (U/L)**	21.0 (15.0-32.5)	20.0 (17.5-27.0)	.889
**Indirect bilirubin (mg/dL)**	0.40 (0.30-0.60)	0.30 (0.20-0.55)	.370
**ADATMS13 activity (%)**	3.0 (3.0-5.0)	3.0 (3.0-7.5)	.787
**ADAMTS13 inhibitor (IU/mL)**	1.7 (0.6-5.8)	1.0 (0.5-8.2)	.925

Continuous variables are summarized as median and interquartile range (25th-75th percentiles).

Bold values indicate *P* < .050.

Among the 11 stroke episodes, 2 were treated with both thrombolysis and mechanical thrombectomy, 1 underwent thrombectomy alone, and the remaining received conservative management for acute stroke. In addition, 6 stroke episodes with low ADAMTS13 activity were treated with plasma exchange (PLEX). Four episodes received 5 PLEX sessions, while the remaining 2 were treated with 4 and 9 sessions, respectively. Rituximab was administered in most episodes (10 of 11), and corticosteroids were used in 5 episodes. Caplacizumab was administered in 2 episodes. Despite treatment with PLEX, corticosteroids, and rituximab, 2 patients failed to achieve ADAMTS13 remission by 6 months. One patient died within 30 days of the stroke, likely secondary to a cardiac arrhythmia. Following recovery from the acute event, 60% of the patients had persistent residual neurologic deficits.

As the awareness of this clinical entity has increased, routine ADAMTS13 testing has been proposed for patients presenting with cryptogenic ischemic stroke, irrespective of prior history of TTP [8], [9]. This study represents the largest reported cohort of patients with ischemic stroke associated with low ADAMTS13 activity in the absence of MAHA. The 10 patients included in the present study largely overlap with those described in a separate manuscript characterizing embolic stroke of undetermined source associated with ADAMTS13 deficiency [10]. Compared with that cohort, the current study excluded 1 patient with positive antiphospholipid antibodies and Libman–Sacks endocarditis and included 2 additional patients who were identified after the recruitment period of the previous study.

In contrast to the initial descriptive report [10], this study focuses on comparative analysis aimed at identifying clinical and laboratory features associated with increased stroke risk. TTP survivors with asymptomatic ADAMTS13 relapse were selected as a control group to facilitate identification of factors associated with increased stroke risk. Compared with controls, patients in the stroke group had a higher prevalence of preexisting vascular comorbidities, including hypertension, cardiac disease, and prior stroke. Evidence from animal models supports a potential pathogenic role for ADAMTS13 deficiency in the development and progression of ischemic stroke. In experimental models of focal cerebral ischemia, ADAMTS13-deficient mice demonstrated significantly larger cerebral infarctions compared with the wild-type mice [11], [12], [13]. These findings may provide a mechanistic explanation for the predominance of large-vessel involvement, particularly middle cerebral artery territory strokes, observed in our stroke patients. In patients with acute stroke, ADAMTS13 activity is also reduced.

Further prospective studies are needed to determine whether low ADAMTS13 activity directly contributes to stroke pathogenesis or is a secondary consequence of the acute stroke [14]. In patients with established risk factors for cerebrovascular disease, reduced ADAMTS13 activity may represent an additional prothrombotic factor that increased susceptibility to ischemic stroke. Patients in the stroke cohort demonstrated significantly higher levels of LDH, blood urea nitrogen, creatinine, and absolute neutrophil counts, along with relatively lower platelet counts, despite otherwise preserved complete blood count parameters and the absence of overt MAHA. A similar pattern was observed in a prior study with isolated ADAMTS13 relapse, in which rituximab therapy was associated with significant increases in platelet counts and reductions in LDH levels, even though these values were within normal ranges prior to treatment [15]. This observation may reflect subclinical endothelial injury or a low-grade microangiopathic process in patients with reduced ADAMTS13 activity [16], even in the absence of clinically apparent MAHA.

There is no currently established consensus regarding optimal management of patients with ischemic stroke associated with severe ADAMTS13 deficiency in the absence of MAHA. In our cohort, treatment was provider-dependent with therapeutic intensity generally guided by the severity of the clinical presentation. Boothby and colleagues have proposed a short course of 5 PLEX sessions to facilitate ADAMTS13 inhibitor clearance in patients presenting with acute stroke [8]. Preemptive rituximab therapy for asymptomatic ADAMTS13 relapse has been standard practice for over a decade [17], [18].

Based on our findings, high-risk patients with ADAMTS13 relapse without hematologic abnormalities—particularly those with significant preexisting medical comorbidities, relatively lower platelet counts, elevated LDH levels, and mild acute kidney injury, as well as Black ethnicity—may benefit from closer laboratory and clinical surveillance, including serial monitoring of ADAMTS13 activity, in addition to the current standard of care. Prompt initiation of rituximab therapy when ADAMTS13 activity declines below 20%, along with consideration of earlier or more intensive preemptive immunosuppressive therapy in selected high-risk patients, may help reduce the risk of ischemic stroke, although this approach requires prospective validation. In addition, counseling high-risk patients regarding the signs and symptoms of stroke and the importance of seeking immediate emergency medical evaluation may facilitate earlier diagnosis and intervention, with the potential to mitigate neurologic injury. Identifying patients at highest risk is critical, as 60% of patients in our cohort experienced persistent neurologic deficits or long-term complications despite recovery.

In summary, ADAMTS13 deficiency represents an underrecognized and potentially modifiable risk factor for cryptogenic stroke. Incorporation of ADAMTS13 testing into the routine stroke diagnostic evaluation should be considered. Early identification and prompt intervention aiming at suppressing inhibitors and restoring ADAMTS13 activity may be critical, as delayed treatment may result in irreversible neurologic injury. Prospective studies are needed to define incidence, refine risk stratification, and establish evidence-based treatment strategies in this rare but clinically consequential condition. In TTP survivors with ADAMTS13 relapse, beyond routine rituximab therapy, early identification of high-risk individuals is crucial to avoid further adverse neurologic outcomes.
